# Investigating alterations associated with heat stress and the recovery of the intestinal barrier using IPEC-J2 as an intestinal epithelial porcine cell model

**DOI:** 10.1038/s41598-026-45755-z

**Published:** 2026-04-01

**Authors:** M.-H. Perruchot, G. Boudry, S. Wiart-Letort, M. Mauro-Vigroux, S. Daré, F. Gondret, M.-L. M. Grundy

**Affiliations:** 1 PEGASE, INRAE, Institut Agro, 35590 Saint Gilles, France; 2 Institut NUMECAN, INSERM, INRAE, Univ Rennes, 35590 Saint Gilles, France

**Keywords:** Biochemistry, Cell biology, Gastroenterology, Physiology

## Abstract

Heat stress (HS) is a growing concern due to global warming, especially for species with limited thermoregulatory capacity. The mechanisms underlying HS effects on digestive functions remain to be elucidated. The present study investigated the impacts of HS (41 °C for 5 days) followed by a recovery phase (37 °C for 5 days) to simulate a heatwave, on porcine intestinal epithelial cells (IPEC-J2) by focusing on barrier defense mechanisms. During HS, cell viability and apoptosis, permeability, tight junction (TJ) proteins, mucin expression and oxidative stress balance were affected as compared to cells staying at 37 °C (TN)(P < 0.05). During the recovery phase, most of the HS-induced alterations were rapidly reversed: apoptosis rates were normalized, paracellular permeability returned to Thermo Neutral (TN) levels, and TJ proteins (ZO-1 and occludin) localization were restored. Antioxidant enzyme abundance and Nrf2 levels were also similar to those in TN cells, but HSP70 abundance decreased below TN levels. Altogether, this study reveals specific adaptive intestinal mechanisms to HS, contributing to partial resilience of the digestive functions under heatwaves.

**Supplementary Information:**

The online version contains supplementary material available at 10.1038/s41598-026-45755-z.

## Introduction

Heatwaves have increased in intensity, frequency and duration during the last decades, with these trends projected to worsen in the next decades^1^. According to the climate science literature, heatwaves correspond to at least 3 or 6 consecutive days where temperature exceeds 90 to 95th percentile of maximum temperature^2^. Hot temperatures trigger adaptation mechanisms of living organisms, especially those with limited thermoregulatory capacity. Homeotherms can maintain relatively constant body core temperature within narrow limits despite wide variations in climatic environment, but can no longer control their body core temperature when ambient temperature increases above the upper critical limit. Among animal species, pigs are particularly vulnerable to high temperatures due to the absence of sweat glands, and they dissipate less than 50% of their heat production by respiratory evaporation^3^. Besides important economic consequences for pig farms^4^, studying heat stress (HS) in this species can offer important cues for anticipating adaptative problems and managing health and welfare of human populations due to the close proximity of pig and humans in term of digestive physiology.

Pig body core temperature rapidly increases when facing acute or chronic heat exposure with a rectal temperature of 39.5 °C after 5 h at 40 °C^5^, and above 40 °C after 20 days at 36 °C^6^. On the contrary, when pigs are exposed to a moderately high temperature during a prolonged period, they first present a phase of increasing body core temperature that then declines to a dynamic steady state. This ‘acclimated’ state corresponds to physiological adaptations, enabling individuals to better tolerate extreme temperatures^7^. However, these variations in body core temperature may transitorily or permanently affect the functioning of internal organs such as the digestive tract^8^. High ambient temperature, particularly above 32 °C, has been shown to disrupt intestinal mucosal homeostasis and impair barrier function, generating malabsorption, ulceration and inflammation^9^.

Among the digestive components, the intestinal epithelium is highly sensitive to environmental stress^10^. It has been demonstrated that heat stress reduced pig intestinal integrity and could disrupt tight junctions (TJ), through an effect on TJ protein expression such as ZO-1 (zonula occludens-1), claudins, and occludin^11^, especially in the jejunum^12^. In addition, HS affected the intestinal mucus production (mucin 2), which is mainly secreted by goblet cells, and provides barrier protection of the intestine^9,13–15^. In addition, HS induced oxidative stress through excessive reactive oxygen species (ROS) production^16^. Redox signaling influences various pathways such as apoptosis, ferroptosis, endoplasmic reticulum stress, inflammation and nuclear factor erythroid-related factor 2 (Nrf2) signaling pathway^17^. Oxidative insult can also disrupt TJ integrity, with an upregulation of occludin protein expression, downregulation of ZO-1, but no effect on claudin-3 levels in Caco-2 cells^17^. This TJ remodeling led to increased intestinal epithelial permeability and subsequent translocation of luminal antigens and endotoxins^18^. Although the impact of HS on gut integrity is increasingly being elucidated, the restoration of intestinal barrier function after temperatures return to normal remains poorly characterized in pigs^19^.

The IPEC-J2 porcine epithelial cells represent a relevant model to study intestinal physiology in growing pigs, as they are cells isolated from the jejunal epithelium of a newborn piglet that display most functions of primary intestinal epithelial cells^20^. To date, several studies have used the porcine intestinal epithelial cell line IPEC J2 as an in vitro model to investigate the effects of heat stress on intestinal barrier function and cell integrity. For example, Tang et al. showed that exposure of IPEC J2 cells to elevated temperatures impaired transepithelial electrical resistance and downregulated tight junction proteins such as occludin and ZO 1, consistent with compromised barrier integrity under heat stress, and that antioxidant supplementation alleviated these effects^15,21^. Similarly, Zhang et al. demonstrated that heat stress decreased cell viability and increased epithelial permeability in IPEC J2 cells, concomitant with reduced expression of tight junction proteins and increased apoptosis, indicating a direct impact of elevated temperature on epithelial integrity^22^. In addition, Cui et al. that acute heat exposure induces barrier dysfunction in IPEC J2 cells via endoplasmic reticulum stress mediated apoptotic pathways^23^. These studies validate the use of IPEC J2 cells to model heat stress induced disruption of the gut epithelial barrier and provide a rationale for employing this cell line to examine the molecular mechanisms underlying our current observations.

The objective of the present study was therefore to investigate the effects of a 5-day HS period followed by a recovery phase designed to mimic a heatwave on IPEC-J2 barrier and defense functions. To do so, IPEC-J2 cells were exposed to high temperatures (41 °C) for 5 days, followed by a period of 5 days at more physiological temperature (37 °C). We then evaluated cell viability and apoptosis, permeability, TJ protein and mucin2 expression and redox status.

## Methods

### Cells and reagents

The IPEC-J2 cell line was obtained from DSMZ (ACC-701, Braunschweig, Germany; no information was provided by the supplier regarding passage). Dulbecco’s Modified Eagle Medium/Ham’s F-12 (DMEM/F12) was bought from Fisher Scientific (Illkirch-Graffenstaden) The cell viability assay, MTS (3-(4,5-dimethylthiazol-2-yl)-5-(3-carboxymethoxyphenyl)-2-(4- sulfophenyl)-2H-tetrazolium), inner salt reagent, was purchased from Promega (Charbonnières-les-Bains, France). DeadEnd Fluorometric terminal deoxynucleotidyl transferase dUTP nick end labelling (TUNEL) System (Promega), lucifer yellow, bicinchoninic acid (BCA) Protein Assay Kit, Alexa Fluor 594-conjugated anti-ZO-1 monoclonal antibody (mouse, ref 339,194, lot XJ359380), anti-MUC2 antibody (rabbit Polyclonal Antibody, reference PA5-119,292), and Alexa Fluor 568-conjugated antirabbit secondary antibody (donkey) were obtained from Thermo Fisher Scientific. Primary anti-Occludin antibody was obtained from AbCam (Ab 216,327, AbCam, Cambridge, United Kindom). Paraformaldehyde was purchased at VWR International (Rosny-sous-Bois, France) and 4–12% SDS–polyacrylamide gels (NuPage 4–12% Bis–Tris, NP0323BOX) were obtained from Invitrogen Life Technology (Berlin, Germany). Polyvinylidene difluoride membranes were purchased at GE Healthcare Bio-Sciences AB, (Uppsala, Sweden). Primary antibodies against catalase (SC-50508), NFR-2 (SC-722), HSP70 (SC370887) and SOD (SC-271014) were purchased at Santa Cruz Biotechnology, (Santa Cruz, CA, USA). The CellROX® Deep Red Flow Cytometry Assay Kit (C10491) was purchased from Invitrogen, Life Technology (Berlin, Germany). All other chemicals, fluorescein isothiocyanate − dextran of 4 kDa, solvents, and reagents including the media for the cell culture were obtained from Merck (Saint Quentin Fallavier, France), unless stated otherwise.

### Experimental treatments

The IPEC-J2 cells were grown in 75 cm^2^ flasks containing DMEM/F12 supplemented with 10% porcine serum (PS) containing 1% penicillin–streptomycin as previously described^24^, but without any antioxidant components. Except for viability assay (see corresponding section), cells were then grown on inserts for experiments. At 80% confluence, between passages 4 and 7, cells were seeded on transwell polyester membrane inserts (0.4 μm pore size, 1.1 cm^2^ surface area), placed in 12-well plates at a density of 1 × 10^5^ cells per cm^2^, and in DMEM/F12 medium supplemented with 5% PS, 1% penicillin–streptomycin. The cells were left to grow in a humidified (95%) incubator under 5% CO_2_ during 2 days for attachment and proliferation, and cells cultures’ homogeneity were checked before HS application. Cells were divided into two conditions: TN for cells grown at the reference temperature (37°C) for 5 or 10 days, and HS for cells grown at high temperature (41°C, heat stress) conditions during 5 days and then placed at 37°C for the following 5 days. This temperature was chosen since the body core temperature of growing pigs can reach a maximal value of 40.0–40.5°C during an ambient heat thermal challenge^3^ and rectal temperature can even rise up to 41.5°C. During cell culture to visualize cells with images were obtained with a Zeiss microscope Axio 1.0 using 20X objective.

The medium was changed every two to three days. The transepithelial electrical resistance (TEER) was measured on days 3, 5, 7 and 10 with an Epithelial Voltohmmeter (EVOM3, Friedberg, Germany). TEER values, in Ω × cm^2^, were calculated as follows:

1 $${\text{TEER }} = \, \left( {{\text{R }} - {\text{ R}}_{0} } \right) \, \times {\text{ A}}$$

where R is the measured resistance (Ω), R_0_ is the blank resistance (insert without cells), and A is the inset effective surface (1.1 cm^2^) as previously described^21^.

### Cell Viability by MTS assay

All cells were seeded (200 μL of 1 × 10^5^ cells/mL) into a 96-well microplate and left to incubate in their corresponding medium (5% PS) under 5% CO2 for 5 days under HS or TN conditions. The medium was removed and the cells were washed with Hank’s Balanced Salts Solution (HBSS). Control wells corresponded to the TN condition. After addition of 100 μL of MTS (dilution of 1:12 from the stock solution ) in each well, the cells were further incubated at 37°C or 41°C for TN or HS conditions, respectively, for 1h. The absorbance was then recorded at 490 nm with a microplate reader (Varioskan Lux, ThermoFisher, Villebon-sur-Yvette, France) as previously described^21^. The cell viability was calculated from the ratio of the absorbance of the HS cells to the absorbance of the TN cells.

### TUNEL Assay

To visualize and quantify DNA fragmentation in apoptotic cells, TUNEL assay was performed. After cell fixation 30 min in 4% paraformaldehyde and 10 min in methanol (1 mL in the insert under a fume cupboard) and 2 washes of 5 min with phosphate-buffer saline (PBS) 1X, 100 µL of 200 ng/μL Proteinase K was applied on inserts for 20 min in oven at 37°C. Cells were then incubated with reagents from the DeadEnd Fluorometric TUNEL System (Promega) according to the manufacturer’s instructions. After the TUNEL reaction was stopped, cells were washed 5 min with PBS 1X and the inserts were removed gently from the well, placed on an identified slide and mounted with 15 µL ProLong Gold Antifade Mountain with DNA Stain 4′,6-diamidino-2-phenylindole (DAPI) per insert^25^. The experiments were repeated three times, on different plates. Each time, two wells were used for each condition 5TN or HS). Images were obtained with a Zeiss Apotome fluorescence microscope using 40X objective (Apotome, Zeiss, France) and analyzed with the ImageJ software. Eight microscopic fields (magnification: 40X; area: 0.14 mm2 per microscopic field) were examined for each sample. The percentage of apoptosis was determined as the ratio of the TUNEL-labeled to the DAPI-labelled cells.

### Intestinal permeability

The FITC-dextran 4 kDa (FD4) diffusion assay was carried out on cells under TN or HS conditions at both D5 and D10 to evaluate transepithelial paracellular transport. The FD4 solution (100 μL at 10 mg/mL in HBSS) was added to the apical compartment (total volume of 1mL). Medium (100 μL) was removed in the basal compartment (total volume of 2mL), and replaced with HBSS, after 15, 30, 60, 120 and 240 min of incubation at 37°C or 41°C (D5) and 37°C (D10) for TN and HS conditions, respectively. Fluorescence intensity was measured using the microplate reader at an excitation wavelength of 495 nm and an emission wavelength of 525 nm. The concentration of FD4 (ng/μL) was determined from a standard curve. These experiments were performed in triplicate. The apparent permeability coefficient of FD4 (Papp, cm.s^–1^) was calculated as follows:

2 $$P_{{{\mathrm{app}}}} = \,V\left( {{\text{Ax C}}_{{\mathrm{i}}} } \right){\text{ x C}}_{{\mathrm{f}}} {\mathrm{T}}$$

where V is the volume in the basal compartment in mL, A is the surface area of the insert, C_i_ the initial concentration of FD4 in the apical compartment, C_f_ the concentration of FD4 in the basal compartment, and T the time in second.

Complementary to the FD4 assay, the passage of lucifer yellow (LY, 521.8 Da) across the epithelial cell monolayers was monitored. The culture medium was removed at D5 and D10, and the monolayers were washed with HBSS. LY (final concentration of 200 μM) was added to the apical compartment containing HBSS. The monolayers were left to incubate at 37°C or 41°C (D5) and 37°C (D10) for TN and HS conditions, respectively, and 100 μL of the basal media was removed at 120 min. The fluorescence was measured at an excitation wavelength of 428 nm and an emission of 540 nm with the microplate reader. The LY concentration, in μM, was determined from a standard curve (0 to 200 μM). Each assay was repeated three time. The Papp of LY was calculated according to the Eq. (2).

### Mucin 2 (MUC2) gene product

The mucin 2 (MUC2) gene production was evaluated using two different techniques: flow cytometry and immunocytochemistry. For flow cytometry, cells were treated with trypsin and rinsed with PBS. About 800,000 cells were placed 30 min at 4°C in PBS containing 0.5% Bovine Serum Albumin (BSA) and incubated with anti-MUC2 antibody, which was then detected using a conjugated secondary antibody goat anti-rabbit Alexa Fluor 488 (A 11,008) during 1h. All data were compared to isotype-matched negative controls. Labeled cells were then analyzed using a MACSQuantR flow cytometer and software (Miltenyi Biotec, Paris, France) and FlowLogic software (Innivai, Australia). A minimum of 20,000 events was acquired for each sample. For immunocytochemistry, after fixation and permeabilization as previously described, the monolayers were incubated with either wheat germ agglutinin or MUC2 polyclonal antibody, both diluted 1:100 in PBS-0.2% BSA^24^ For MUC2, the samples were then incubated with antirabbit secondary antibody conjugated with Alexa Fluor 568, diluted 1:300 in PBS-0.2% BSA^24^. Images were obtained with a Zeiss Apotome fluorescence microscope using 40X objective.

### Tight junction (TJ) protein immunocytochemistry

In TN and HS conditions, the apical and basolateral compartment of the cells were emptied, and the monolayers were fixed 30 min in 4% paraformaldehyde, then with methanol, washed twice in PBS solution and permeabilized with Triton X100 and washed twice again with PBS as previously described^24^ The monolayers were then incubated for 1.5h at 37°C with a primary anti-ZO-1 antibody conjugated with Alexa Fluor 594 (100 μL) or a primary anti-Occludin followed by an anti-rabbit Alexa 488, diluted 1:100 or 1:400 respectively, in PBS-0.2% BSA. The samples were mounted on microscopy slides with 15 μL of ProLong Gold Antifade Mountain with DAPI. The fluorescence intensity for ZO-1 and Occludin was analyzed using the ImageJ software. The immunocytochemistry experiments were repeated three times, on different plates, incubated several weeks apart. For each time two wells were used for each condition, taking 8 images per well.

### Western blot analysis for TJ and antioxidant targets

Total proteins were extracted with Radioimmunoprecipitation buffer (Fisher Scientific, Illkirch-Graffenstaden, France) in cells under TN or HS conditions. Protein concentrations were determined using a BCA Protein Assay Kit. The proteins were separated by SDS-PAGE on 4–12% SDS–polyacrylamide gels (NuPage 4–12% Bis–Tris), transferred to polyvinylidene difluoride membranes and incubated overnight with primary antibodies against ZO-1, CAT, NRF-2, HSP70 or SOD (dilution 1:200 for each). Horseradish peroxidase-conjugated secondary antibody was used at 1:2,500, and chemiluminescence was visualized using an ECL Kit and an ImageQuant LAS4000 Biomolecular Imager digital imaging system (GE Healthcare, Velizy-Villacoublay, France). All original plots are available as supplementary information.

### Antioxidant enzyme activities and total antioxidant capacity

In our study, H₂O₂ was used as a well-established in vitro model to mimic the oxidative component of HS, since thermal stress in intestinal epithelial cells is known to generate ROS and cause oxidative damage. This approach allows us to specifically evaluate the cellular response to oxidative stress, which is a major downstream effect of heat stress in epithelial cells. The applied H₂O₂ concentrations were selected based on previous studies in intestinal epithelial cells and preliminary experiments to identify conditions that induce measurable oxidative stress without causing excessive cytotoxicity. For example, Hsu et al., 2025 used H₂O₂ (200-600μM) cells to induce oxidative stress and assess barrier function and antioxidant responses^26^ and Wang et al., treated cells with H₂O₂ to 0–500 μM treatment in intestinal epithelial cells^27^. These studies demonstrate that H₂O₂ concentrations in this range reliably generate oxidative stress in intestinal epithelial models without excessive cell death.

The activities of antioxidant enzymes catalase (CAT), superoxide dismutase (SOD) and glutathione peroxidase (GPX) and glutathione reductase (GRX) participating to the redox system regenerating the main non-enzymatic antioxidant glutathione were determined in TN and HS cell lysates after homogenization in PBS buffer (pH 7.2), cellular suspension (approx.4.10^6^cells/ml PBS) were sonicated following a defined protocol of fractionated sonication (5 cycles/3^*^10s per cycle). The homogenates were then centrifuged for 10 min at 8,000 g at 4°C, and the supernatant was collected. The CAT activity was measured by spectrometry at 240 nm using an Uvikon Bio-Tek apparatus (Secomam, Alès, France) following the decrease in hydrogen peroxide (H_2_O_2_) concentration at 25°C as previously described^28^. Total SOD activity responsible for the elimination of cytotoxic active oxygen by catalyzing the dismutation of the superoxide radical to O_2_ and H_2_O_2_, was measured at 450 nm by the inhibition of the xanthine/xanthine oxidase-mediated oxidation of cytochrome-c, using a dedicated kit (19,160 SOD; Sigma Aldrich, St. Louis, MO, USA) with a SOD standard from bovine erythrocytes (S7571-15KU; Sigma Aldrich) and the microplate reader^24^. Activities of GSH-Rx and GSH-Px were assessed spectrophotometrically at 37°C on the Gallery plus analyzer (Thermo Electron, Courtaboeuf, France) by following NADPH decrease at 340 nm absorbance^29^. All enzyme activities were expressed as units/mg of proteins. Protein content was used to calculate specific enzyme activity (units/mg protein). The ratio of GPX to GRX was also calculated.

Total antioxidant activity was estimated in TN and HS cell lysates by the ferric reducing antioxidant power (FRAP) method, which is based on the reduction of complexes of 2,4,6-tripyridyl-s-triazine (TPTZ) with ferric chloride hexahydrate producing blue ferrous complexes. The deviation of absorbance was measured at 595 nm at 37°C for 30 min in darkness^30,31^ by using the Gallery plus analyzer and values are expressed as equivalent TROLOX per mL of supernatants.

### ROS quantification

To stimulate ROS production, TN or HS cells were incubated for 1h at 37°C or 41°C with or without 200 or 500 μM H_2_O_2_ diluted in cell culture media in a 96-well black microplate. Cells were then stained for 30 min with 100 nM CellROX red reagent. A viability marker was added to differentiate dead from alive cells after H_2_O_2_ stimulation (Fixable Viability Dye Efluor 780, 1/10,000 [vol/vol], Invitrogen, Life Technology, Berlin, Germany). The fluorescence resulting from CellROX® Oxidative Stress Reagents was measured by flow cytometry on MACSquant cytometer (Miltenyi Biotec, Paris, France) at 640/665 nm and dead cells were excluded with blue SYTOX at 444/480 nm. A total of 20,000 cells were acquired using a MACSQuant Analyzer 10 cytometer (Miltenyi Biotec, Paris, France). The results were analyzed with FlowLogic software (Innivai, Australia).

### Statistical analysis

The data analysis was conducted using GraphPad Prism v9.0.0 (for Windows 64-bit, GraphPad Software, San Diego, CA, USA). For all tests, the significance level was set at P < 0.05 and all data were expressed as means ± standard error of the mean (SEM). The differences between the two treatments (TN vs HS) were assessed by either two-way ANOVA (time and culture condition as fixed effect) followed by Tukey’s post hoc test.

## Results

### Cell morphology, viability and apoptosis

**Fig. 1:**
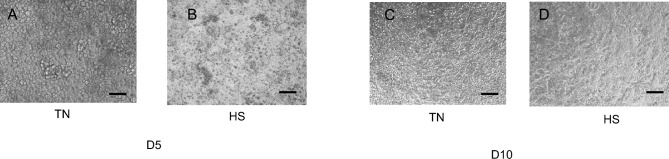
Representative images of IPEC J2 cells cultured at 37°C (TN) during 10 days or at 41°C (HS) during 5 days and then returned back to 37°C during 5 days. Phase contrast images were obtained in HS and TN conditions at day (D) 5 (**A**, **B**) or D10 (**C**, **D**). Black bar stands for 50 µm.

**Fig. 2:**
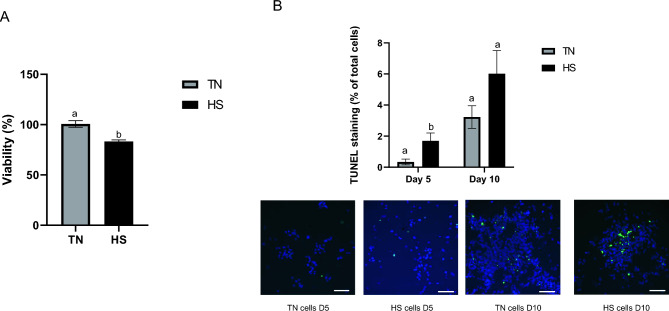
Cell viability and apoptosis of IPEC J2 cells cultured at 37°C (TN) during 10 days or at 41°C (HS) during 5 days and then returned back to 37°C during 5 days. Viability of IPEC-J2 cells was estimated by MTS assay at day 5 of culture (**A**). TUNEL staining percentage was obtained at day 5 (D5) and day 10 (D10) and representative images are shown (**B**). TUNEL nuclei in green and DAPI counterstaining of nuclei in blue. Scale bar = 100 μm. The values are means + /- SEM of 3 replicates in the different conditions and times. Different letters indicate significant difference (P < 0.05).

HS cells visually displayed more nuclear condensation and multiple cell rounding than cells cultured at 37°C (TN), as assessed by microscopy image examinations at D5 and D10 (Fig. 1). Cell viability decreased slightly between HS and TN conditions when evaluated at D5 (Fig. 2A) (P < 0.05). Apoptotic cell level estimated by TUNEL staining was greater (P < 0.05) in HS cells compared to TN cells at D5, but there was no difference between HS and TN conditions at D10 (Fig. 2B).

### Intestinal epithelial cell permeability

**Fig. 3:**
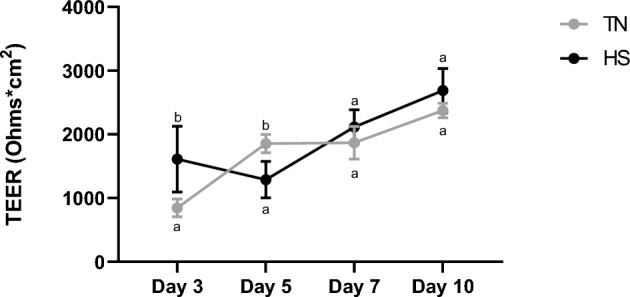
Time course evolution of TEER of IPEC-J2 cells cultured at 37°C (TN) during 10 days or at 41°C (HS) during 5 days and then returned back to 37°C during 5 days. Data are presented as means + /- SEM of three replicates at each time point. The effects of the conditions (TN or HS) and time were analyzed using two-way ANOVA followed by Tukey’s post-hoc test. Different letters indicate significant difference (P < 0.05)

**Fig. 4:**
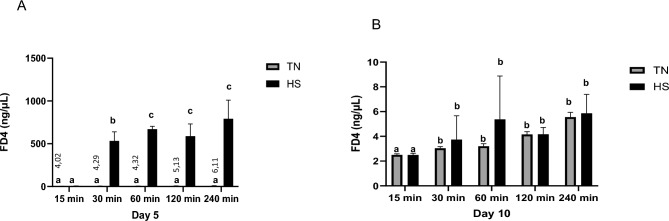
Time course evolution of the passage of FITC-Dextran 4kDa (FD4) across IPEC-J2 monolayer cultured at 37°C (TN) during 10 days or at 41°C (HS) during 5 days and then returned back to 37°C during 5 days. Data are presented as means + /- SEM of three replicates at each time point. The effects of the conditions (TN or HS) and time (min) were analyzed using two-way ANOVA followed by Tukey’s post-hoc test at day 5 (D5, (**A**)) and day 10 (D10, (**B**)). Different letters indicate significant difference (P < 0.05).

**Fig. 5:**
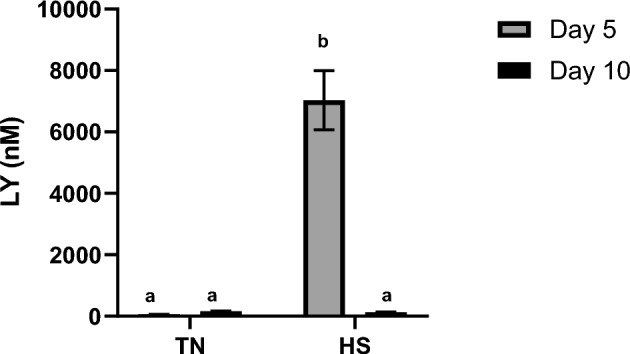
Passage of lucifer yellow (LY) across IPEC-J2 monolayer cultured at 37°C (TN) during 10 days or at 41°C (HS) during 5 days and then returned back to 37°C during 5 days. Data are presented as means + /- SEM of three replicates at each time point. The effects of the conditions (TN or HS) and time (D5 or D10) were analyzed using two-way ANOVA followed by Tukey’s posthoc test. Different letters indicate significant difference (P < 0.05).

Time course variations in TEER were monitored at D3, D5, D7 and D10 (Fig. 3). The TEER was significantly higher at D3 in HS condition (P < 0.05) as compared to TN condition (Fig. 3). Upon return to 37°C from D5 onwards, TEER dramatically increased in HS cells but remained stable in TN cells, so that at D10, TEER was similar in HS cells to TN cells (Fig. 3). Indeed, at D5, paracellular permeability, assessed by the passage of FD4 during 4h (Fig. 4A and B) increased during the first hour in HS cells then remained stable over time. On the contrary, paracellular permeability did not change in TN cells. Overall, the passage of FD4 was higher in HS cells than in TN cells at D5 (Fig. 4A). At D10, paracellular permeability increased during the 4h of the assay in both TN and HS cells (Fig. 4B), with no difference between TN and HS cells at any time point. These results on paracellular permeability were further confirmed using a smaller probe, lucifer yellow, since LY passage across the cell monolayer was higher (P < 0.05) at D5 in HS compared to TN cells (Fig. 5).

### Tight junction protein and mucin production

**Fig. 6:**
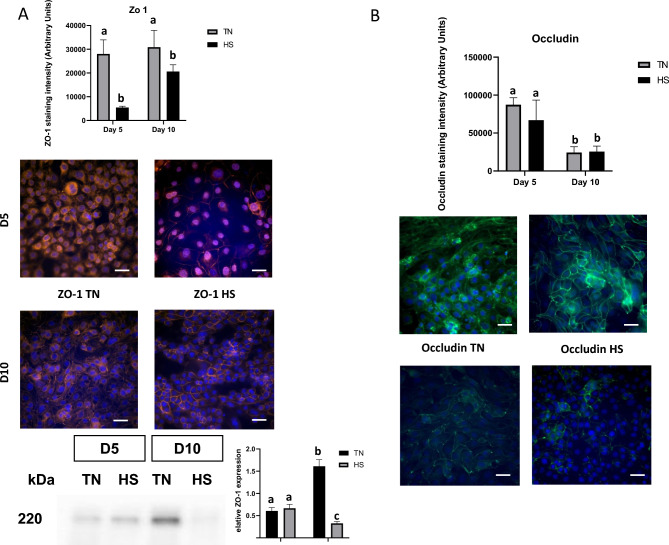
ZO-1 and Occludin immunostaining in IPEC-J2 monolayer cultured at 37°C (TN) during 10 days or at 41°C (HS) during 5 days and then returned back to 37°C during 5 days. Representative western blot of ZO-1 protein expression. The relative abundance of the protein was quantified by a molecular imager for immunoluminescence. Fluorescence quantification a representative image of ZO-1 (**A**) and Occludin (**B**). ZO-1 immunostaining in red, Occludin immunostaining in green and DAPI counterstaining of nuclei in blue. Scale bar = 50 μm. Experiments have been performed three times. Data are presented as mean and SEM. The effect of the treatment (TN or HS) and time were analyzed at Day 5 and Day 10 using two-way ANOVA followed by Tukey’s posthoc test. Different letters indicate significant difference (P < 0.05).

Compared to TN condition, the expression levels of ZO-1 were lower in HS condition at D5 (P < 0.05, Fig. 6A), whereas that of occludin was similar. At D10, the expression levels of ZO-1 were also lower in HS condition and occludin did not differ between HS and TN conditions (Fig. 6 A and B). Moreover, for the TN condition, ZO-1 was mostly located in the cytosol at D5, and then trafficked to the TJ at D10, which likely indicates epithelial barrier maturation. In the HS condition, there was almost no ZO-1 production at D5, whereas protein quantity was increased at D10; however, ZO-1 was localized in the cytosol rather than to the membrane. The determination was based on the subcellular localization pattern of ZO-1 observed by immunofluorescence staining. At D5 under TN conditions, ZO-1 signal was predominantly diffuse and localized within the cytosol, whereas at D10, ZO-1 was mainly concentrated at the cell–cell borders, showing a continuous junctional staining pattern characteristic of tight junction formation. Western blot of ZO-1 confirmed the presence of ZO-1 at 220 kDa (Fig. 6 A), the expression of ZO-1 didn’t change at D5 between TN and HS, at D10 ZO-1 expression increased in TH and decreased significantly in HS cells (p < 0.05) (Fig. 6 A). At D5 ZO-1 protein Both proteins showed progressive increases in expression as the cells differentiated while mRNA expressions were not significantly different (Fig. 3A). We also assessed MUC2 expression as another epithelial barrier defense mechanism (Fig. 6). The percentage of MUC2 positive cells assessed by flow cytometry was reduced at both D5 and D10 in HS cells compared to TN cells (P < 0.05, Fig. 6A and B). These results were confirmed by immunocytochemistry that showed a lower number of MUC2-positive cells in HS condition than in TN condition at both times (P < 0.05, Fig. 6C).

### Cell antioxidant capacities

**Fig. 7:**
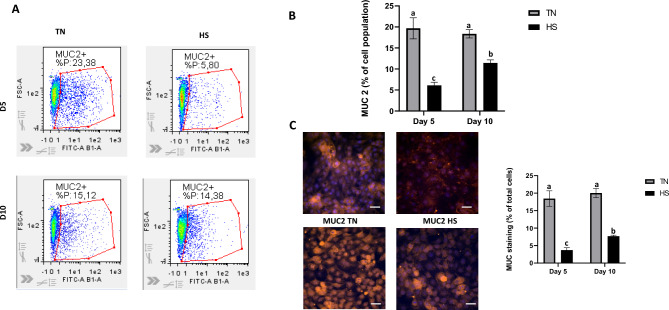
MUC-2 expressing cells in IPEC-J2 monolayer cultured at 37°C (TN) during 10 days or at 41°C (HS) during 5 days and then returned back to 37°C during 5 days. (**A**) Gated MUC2 + cells were set after exclusion of died cells and isotype control as internal control. (**B**) Percentage of MUC2 + cells of IPEC J2 cells at D5 and D10 (**C**) Muc2 immunostaining quantification and representative images at day 5 and day 10. Muc2 immunostaining in red and DAPI counterstaining of nuclei in blue. Scale bar = 50 μm. Experiments were performed three times Data are presented as mean and SEM. The effect of the treatment (TN or HS) and time were analyzed at Day 5 and Day 10 using two-way ANOVA followed by Tukey’s posthoc test. Different letters indicate significant difference (P < 0.05).

**Fig. 8:**
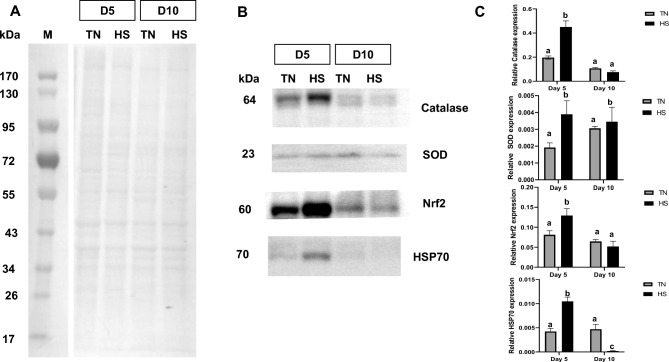
Abundances in antioxidant proteins measured in IPEC-J2 cultured at 37°C (TN) during 10 days or at 41°C (HS) during 5 days and then returned back to 37°C during 5 days. The cells at day 5 (D5) or day 10 (D10) were lysed for total proteins extraction which were then separated by SDS-Page. Gels were stained by Coomassie blue (**A**) for a control of procedure or blotted with monoclonal antibodies (**B**) against Catalase, superoxide dismutase (SOD), Nrf-2 or heat-shock protein-70 (HSP70). Representative images of the corresponding bar are shown. The relative abundance of each protein was quantified by a molecular imager for immunoluminescence (**C**). Data are presented as means + /- SEM of three replicates at each time point. The effects of the conditions (TN or HS) and time (D5 or D10) were analyzed using two-way ANOVA followed by Tukey’s posthoc test. Different letters indicate significant difference (P < 0.05).

**Fig. 9:**
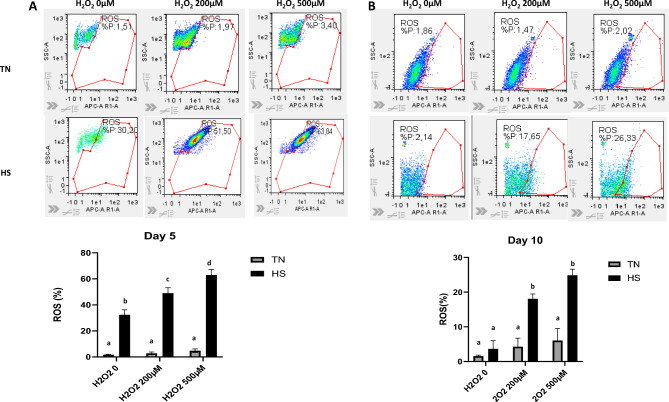
ROS production in IPEC-J2 cultured at 37°C (TN) during 10 days or at 41°C (HS) during 5 days and then returned back to 37°C during 5 days. Died cells were excluded, and ROS production was measured using an isotype control as internal control, at day 5 (D5), (**A**) or day 10 (D10), (**B**) after addition of H202 (0: basal state; 200 or 500 nM). Representative images are shown. For charts, data are presented as means + /- SEM of three replicates at each time point. The effects of the conditions (TN or HS) and H_2_O_2_ doses (0, 200, 500) were analyzed using two-way ANOVA followed by Tukey’s posthoc test. Different letters indicate significant difference (P < 0.05).

To evaluate cell antioxidant capacities, the abundances of CAT, SOD, Nrf2 and HSP70 proteins (Fig. 7) in TN and HS cells were studied by western-blot analyses (Fig. 8 B-C). At D5, all these proteins (P < 0.05) had a greater abundance in HS cells compared to TN cells. At D10, HSP70 protein abundance was markedly lower in HS cells (P < 0.05, Fig. 8 B-C) compared to TN cells. On the opposite, the CAT, SOD and Nrf2 abundances did not differ between HS and TN conditions at D10. When evaluated at the enzyme activity level (Table 1), there were no significant differences between HS and TN cells at D5 and D10 for SOD and GRX enzymes. On the opposite, CAT activity was decreased at D10 in HS cells (P < 0.05) whereas GPX activity was increased in HS cells as well as in TN cells at D10 compared to D5 (Table 1). Total antioxidant capacity (FRAP) was higher in HS condition than in TN condition at D10 (Table 1). Finally, ROS production was measured in D5 and D10 cells after stimulation by H_2_O_2_ at different concentrations (0, 200 or 500 nM) for 1h. At D5, basal ROS production was higher in HS cells than in TN cells (P < 0.05, Fig. 9A). Addition of H_2_O_2_ stimulated ROS production in HS cells, but not in TN cells (P < 0.05). At D10, the basal level of ROS did not differ between HS and TN cells, whereas addition of H_2_O_2_ stimulated ROS production in HS cells but not in TN cells (Fig. 9B, P < 0.05).

**Table 1:** Activities of antioxidant enzymes and total antioxidant capacity in IPEC-J2 cultured at 37 °C (TN) during 10 days or at 41 °C (HS) during 5 days and then returned back to 37 °C during 5 days.

Variables	Day 5		Day 10		*SEM*	*p*-Value	
	TN	HS	TN	HS		Time effect	Temperature effect
Catalase U/mg prot	21.7	28.3	19.0	11.1	6.24	0.04	0.25
SOD U/mg prot	5.24	6.19	4.92	6.97	0.89	0.99	0.99
GRX U/mg prot	57.2	65.1	67.9	71.5	9.42	0.83	0.25
GPX U/mg prot	3.89	5.75	10.3	13.0	4.12	0.03	0.39
GPX/GRX	0.097	0.23	0.39	0.27	0.13	0.29	0.39
FRAP µg eqTROLOX/ml	17.7	23.1	16.0	22.3	3.02	0.83	**0.03**

Values are means ± SEM; n = 3.

## Discussion

The originality of this study was to investigate the effects of prolonged heatwave 5 days of heat stress followed by 5 days at physiological temperature) on the intestinal defense and barrier features using IPEC-J2 cells as a model. Culturing IPEC J2 cells in porcine serum has been shown to yield monolayers with morphological and functional features closer to native porcine jejunal epithelium than when using fetal bovine serum, including appropriate barrier characteristics and transporter profiles^24,32^.

We observed lower TEER under heat stress, which was associated to increased paracellular permeability, as demonstrated by the increased passage of FD4 or LY in IPEC-J2 cells exposed to 41 °C during 5 days compared to cells cultured at 37 °C. An increased transepithelial passage of HRP in 43 °C-treated T84 cells during 1 h compared with control (37 °C) conditions has also been observed^33–35^. Intestinal barrier function relies on several parameters, including a tight regulated balance between cell proliferation, viability and apoptosis. We observed an increased apoptosis of cells exposed to heat condition but no effect on cell viability at the end of the heat stress period (D5), assessing cell viability at D10 could be interesting to determine the extent of cellular damage, the threshold between adaptive and lethal responses. This imbalance between preserved cell viability and increased apoptosis in HS vs TN conditions at D5 likely influences intestinal epithelial turnover, which may in turn be responsible for the altered barrier integrity observed under HS^36,37^. The mechanical barrier of the intestine maintained by TJ proteins is another critical element of the intestinal barrier. Tight junctions are protein complexes composed of transmembrane (e.g., claudins and occludin) and scaffolding proteins (e.g., ZO-1), which anchor transmembrane proteins to the actin cytoskeleton^18^. The type of proteins, their localization in the TJ and their phosphorylation state collectively modulates paracellular transport across the intestinal epithelium. In our study, exposure to heat down-regulated the expression of ZO-1 after 5 days, but had no significant effect on occludin expression. Other reports have described TJ disruption under heat conditions using IPEC-J2 cultured for 24 h at 42 °C or 41.5 °C respectively^21,38^, showing intestinal barrier dysfunction and TJ redistribution. For example, claudin-4 network was disrupted by heat stress applied to IPEC-J2 after 24 h at 41.5 °C^39^. However,^40^ did not observe any heat stress-induced response on TJ proteins. The difference observed in the response to HS conditions between TJ proteins, ZO-1 and occluding, can be explained by their localization: ZO-1 binds to other proteins and forms a scaffold or interacts with specific transmembrane proteins to anchor them to the cytoplasm, whereas occludin can relocate from the apical edge and appears in cytoplasmic granules or be internalized by endocytosis. Since ZO-1 is a well-established tight junction-associated scaffolding protein that relocates to the apical junctional complex during epithelial polarization^40^. Our findings indicate that tight junction modulation occurs in a maturation-dependent manner and is restricted to fully differentiated monolayers. We therefore conclude that barrier alterations observed at D10 are associated with changes in ZO-1 expression and junctional organization, whereas at D5 the epithelial layer is not yet sufficiently mature to support functional tight junction remodeling.

Beside TJ, the mucus layer is also an integral component of the protective system established by intestinal epithelial cells. Mucins constitute a selective diffusion barrier permeable to small molecules like nutrients but not to macromolecules^41^. MUC2 expression in IPEC-J2 cells has also been reported under specific culture conditions^32^, with recent work demonstrating detectable MUC2 protein and mRNA in these cells when cultured in porcine serum with agitation or air–liquid interface^42^. In this study, MUC2 expression was significantly altered in HS cells as compared to TN cells at D5 and D10. This suggests that heat stress impaired intestinal barrier protection. This may be attributed to goblet cell loss or dysfunction as many goblet cells died during stress phases^43^. Epigenetic and transcriptional suppression such as stress-responsive factors (activating transcription factor 6 (ATF6) and X-box binding protein-1 (XBP-1) or C/EBP homologous protein (CHOP) can also repress MUC2 transcription for a long time^44^.

Finally, heat stress is also known to induce oxidative stress, due to a higher ROS production^45^. In our study, ROS productions at the basal state and after H_2_0_2_ stimulation were higher in HS condition compared to TN condition. The higher abundance of antioxidant-related proteins in the HS group at D5 likely reflects a stress-induced adaptive response rather than intrinsically superior antioxidant capacity. HS is known to increase intracellular ROS) production, which activates redox-sensitive signaling pathways, particularly the Nrf2 pathway. Upon activation, Nrf2 translocated to the nucleus and promotes the transcription of antioxidant enzymes such as SOD and CAT. Similarly, HSP70 is upregulated as part of the cellular protective response to thermal and oxidative stress. Therefore, the increased protein abundance observed in HS cells most likely represents a compensatory protective response to elevated oxidative stress. While this upregulation suggests activation of antioxidant defense mechanisms, it does not necessarily indicate that the overall antioxidant capacity is better than in TN cells^45^. Instead, it suggests that HS cells are under greater oxidative challenge and are mounting a defensive response. Moreover, despite the increased protein abundance of some of the antioxidant enzymes (catalase, SOD) after 5 days in cells under heat conditions, their specific activities were rather similar in those cells as compared to those cultured at 37 °C (TN condition). Niu et al. (2024) observed increased ROS production in IPEC-J2 under HS (42 °C vs 37 °C) for 1.5 h, but deceased SOD protein^46^. Differences in the duration of heat exposure may explain differences between the two studies, with different adaptation mechanisms in chronic vs acute heat exposure. In the current study, both inducible HSP70 expression, playing a role in adverse stress resistance^10^, and Nrf2, a transcription factor that regulates the cellular defense against oxidative insults, were activated after 5 days under HS conditions. This could explain the elevation of ROS production in IPEC-J2 cells and their increased susceptibility to H_2_O_2_ stimulation. Similarly, HSP70 was dramatically upregulated in heat-stressed pig intestines^47,48^. HSP70 plays a major role in the process of heat tolerance and is even considered the standard index to measure the degree of HS^49^. In our experimental model, we focused on HSP70 because it represents the canonical readout of heat shock response activation nevertheless analysis of HSP25/27 could provide complementary information^47^, HSP70 induction alone is generally considered sufficient to confirm activation of the heat stress pathway. Noteworthy, the cellular oxidative stress observed here in IPEC-J2 cells cultured at 41 °C may also account for the disrupted barrier function observed in HS condition since ROS can damage TJ proteins or induce mitochondrial dysfunction, leading to reduced energy available for barrier maintenance^37,50^.

When HS cells were returned to 37 °C for 5 days, thus at the temperature maintained over time in TN cells, there were no more difference in apoptosis rate nor in epithelial paracellular permeability (FD4 and LY passages) between HS and TN cells. We even observed an increase in TEER during the recovery period for HS cells. The presence and localization of TJ proteins (ZO-1 and occludin), the abundance of antioxidant enzymes (CAT, SOD) and of Nrf2 also returned to those observed in TN cells. However, not all the defense mechanisms were restored. Indeed, although MUC2 was greater at D10 compared to D5 in HS cells, it did not reach the level measured in TN cells. Similarly, whereas ROS levels were similar at the basal state in HS and TN cells at day 10, the H_2_0_2_-induced ROS levels were o still greater in HS than TN cells at this time. Interestingly, the HSP70 abundance was even lower in HS cells than in TN cells after the recovery phase, which could provide an advantage for the cells when experiencing another heatwave. Although monitoring these events at D5 and D10 did not allow us to evaluate the precise kinetics of epithelial function restoration, our results suggest resilience of intestinal cells after heat stress. In vivo studies have also demonstrated that intestinal integrity was affected drastically at day 2 under HS conditions, but pigs started to recover most of their growth rate and intestinal integrity by day 7^51^. Moreover, cellular repair associated with a rapid regeneration of epithelial cells and basic barrier integrity has been observed very rapidly within 3 h after HS in vivo^19^.

In conclusion, using the IPEC-J2 model of pig intestinal epithelium, we show that HS damaged intestinal integrity. HS increased cell apoptosis, disrupted tight junctions, reduced mucus production, and raised oxidative stress, leading to higher paracellular permeability and lower TEER. After returning to normal temperature, most barrier and antioxidant functions recovered quickly. However, persistent reductions in MUC2 expression and heightened oxidative sensitivity indicate that some protective mechanisms are only partially restored.

## Supplementary Information

Supplementary Information.

## Data Availability

All the data are available on: 10.57745/A0ENFT. 10.57745/A0ENFT
