# Supplementary material for: Investigating alterations associated with heat stress and the recovery of the intestinal barrier using IPEC-J2 as an intestinal epithelial porcine cell model
Source: Sci Rep. 2026 Apr 1;16:15453. doi: 10.1038/s41598-026-45755-z (PMC13184058; doi:10.1038/s41598-026-45755-z)
Supplement: Supplementary file 1 — Supplementary Information. [file 41598_2026_45755_MOESM1_ESM.pptx]

## Slide 1
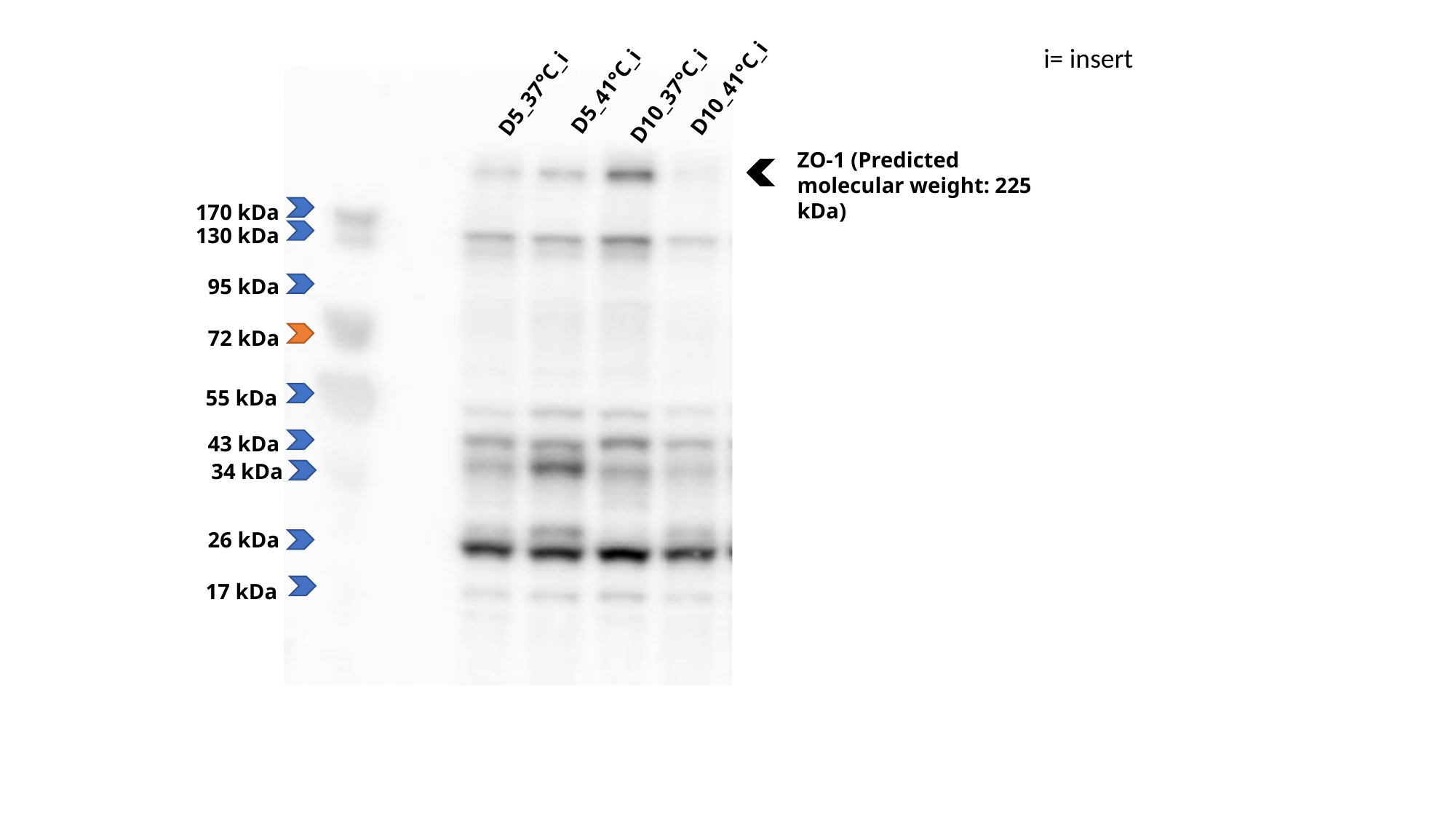

D10_41°C_i
D5_41°C_i
D5_37°C_i
D10_37°C_i
i= insert
ZO-1 (Predicted molecular weight: 225 kDa)
170 kDa
130 kDa
95 kDa
72 kDa
55 kDa
43 kDa
34 kDa
26 kDa
17 kDa

## Slide 2
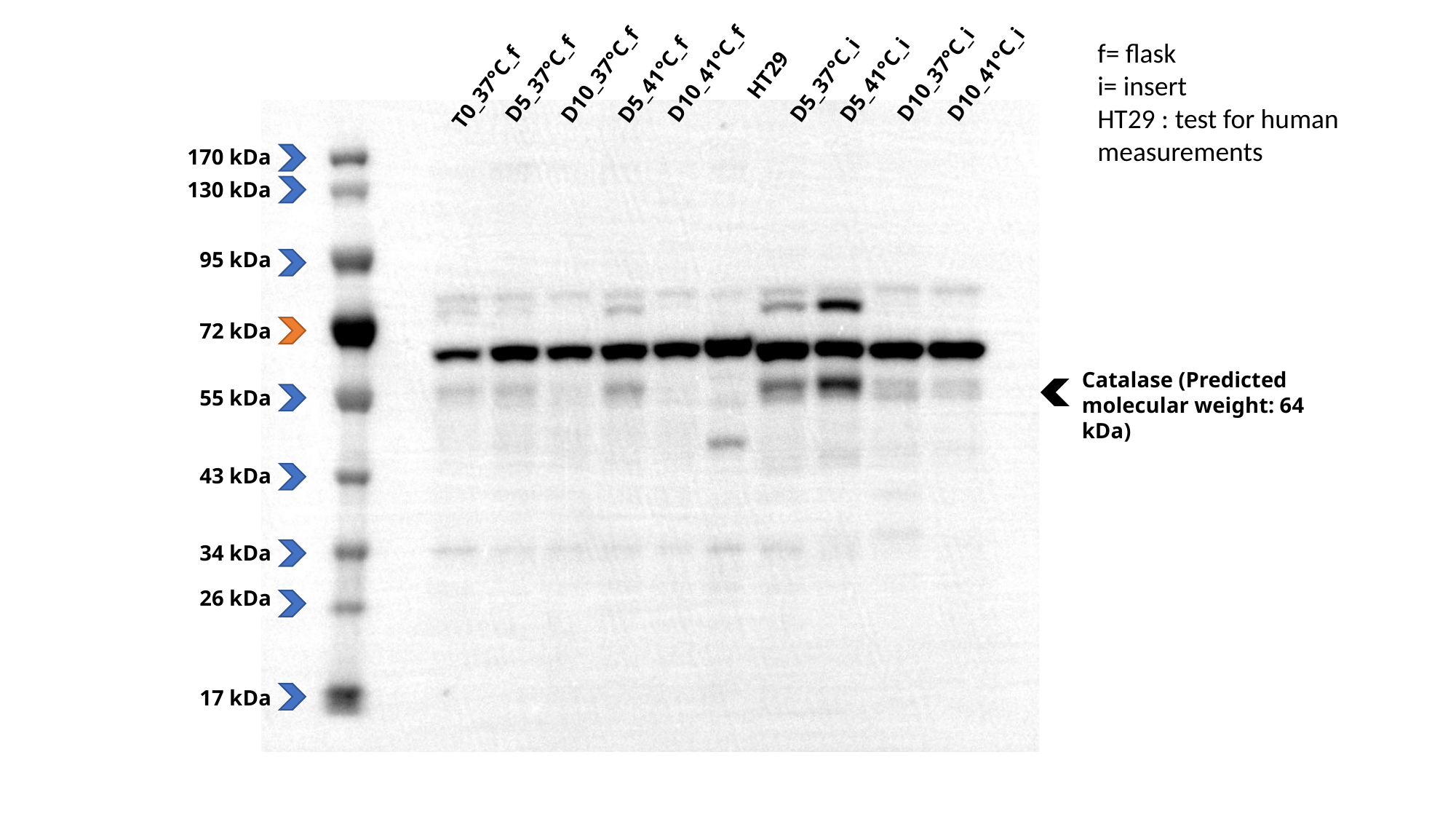

D10_41°C_f
D10_37°C_i
D10_41°C_i
D10_37°C_f
HT29
D5_37°C_f
D5_37°C_i
D5_41°C_f
D5_41°C_i
T0_37°C_f
f= flask
i= insert
HT29 : test for human
measurements
170 kDa
130 kDa
95 kDa
72 kDa
55 kDa
43 kDa
34 kDa
26 kDa
17 kDa
Catalase (Predicted molecular weight: 64 kDa)

## Slide 3
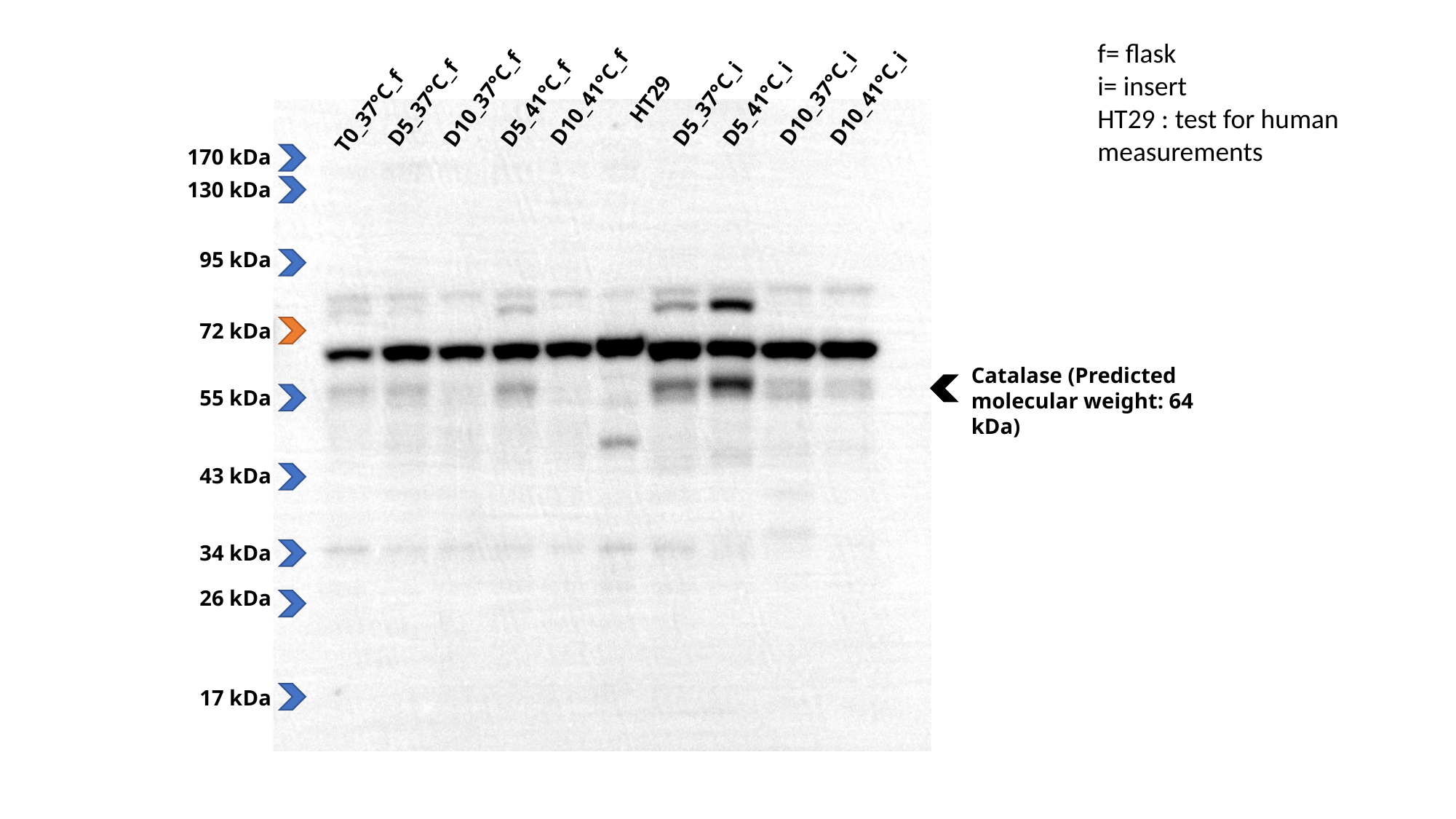

f= flask
i= insert
HT29 : test for human
measurements
D10_41°C_f
D10_37°C_i
D10_41°C_i
D10_37°C_f
HT29
D5_37°C_f
D5_37°C_i
D5_41°C_f
D5_41°C_i
T0_37°C_f
170 kDa
130 kDa
95 kDa
72 kDa
55 kDa
43 kDa
34 kDa
26 kDa
17 kDa
Catalase (Predicted molecular weight: 64 kDa)

## Slide 4
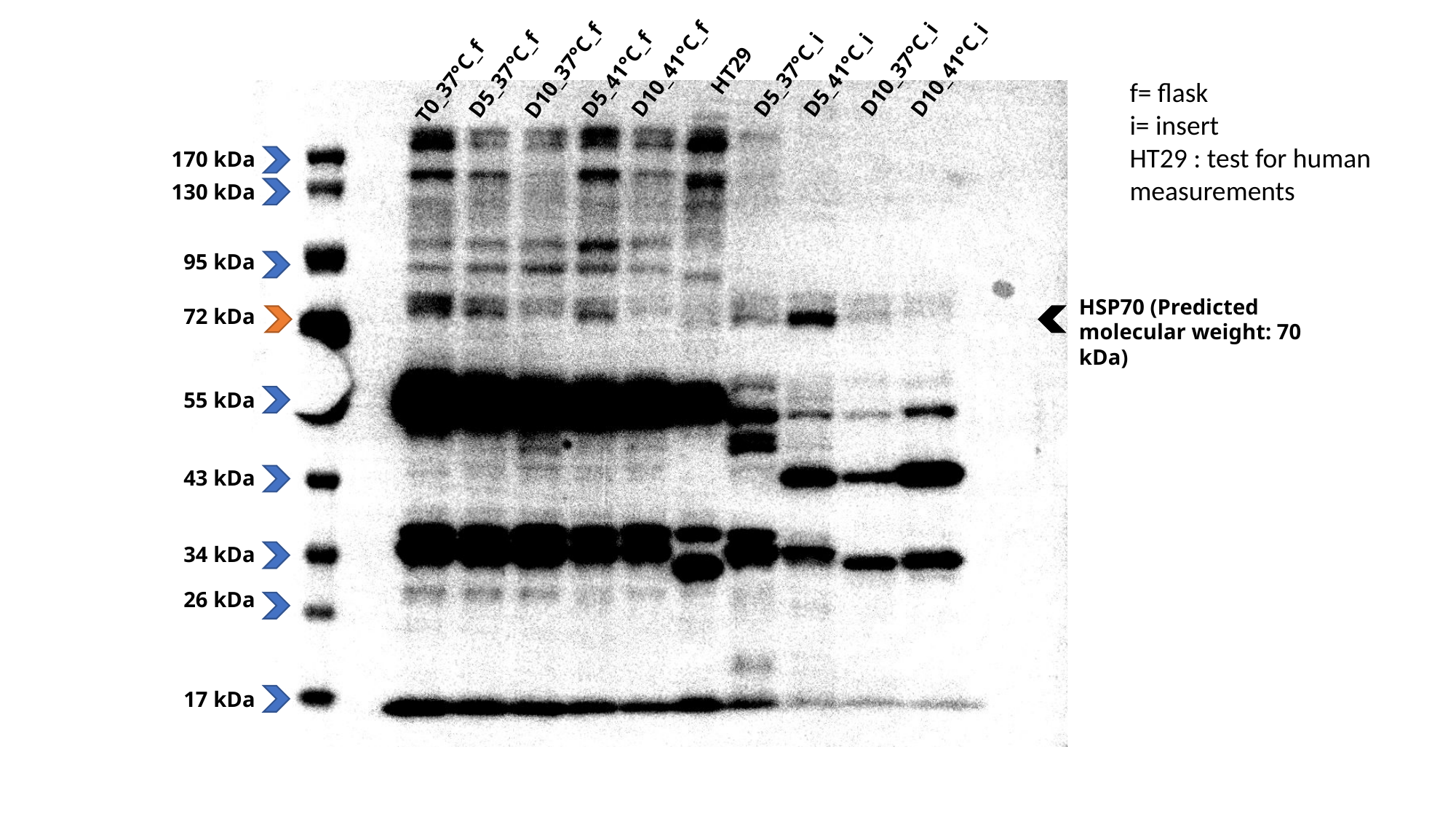

D10_41°C_f
D10_37°C_i
D10_41°C_i
D10_37°C_f
HT29
D5_37°C_f
D5_37°C_i
D5_41°C_f
D5_41°C_i
T0_37°C_f
f= flask
i= insert
HT29 : test for human
measurements
170 kDa
130 kDa
95 kDa
72 kDa
55 kDa
43 kDa
34 kDa
26 kDa
17 kDa
HSP70 (Predicted molecular weight: 70 kDa)

## Slide 5
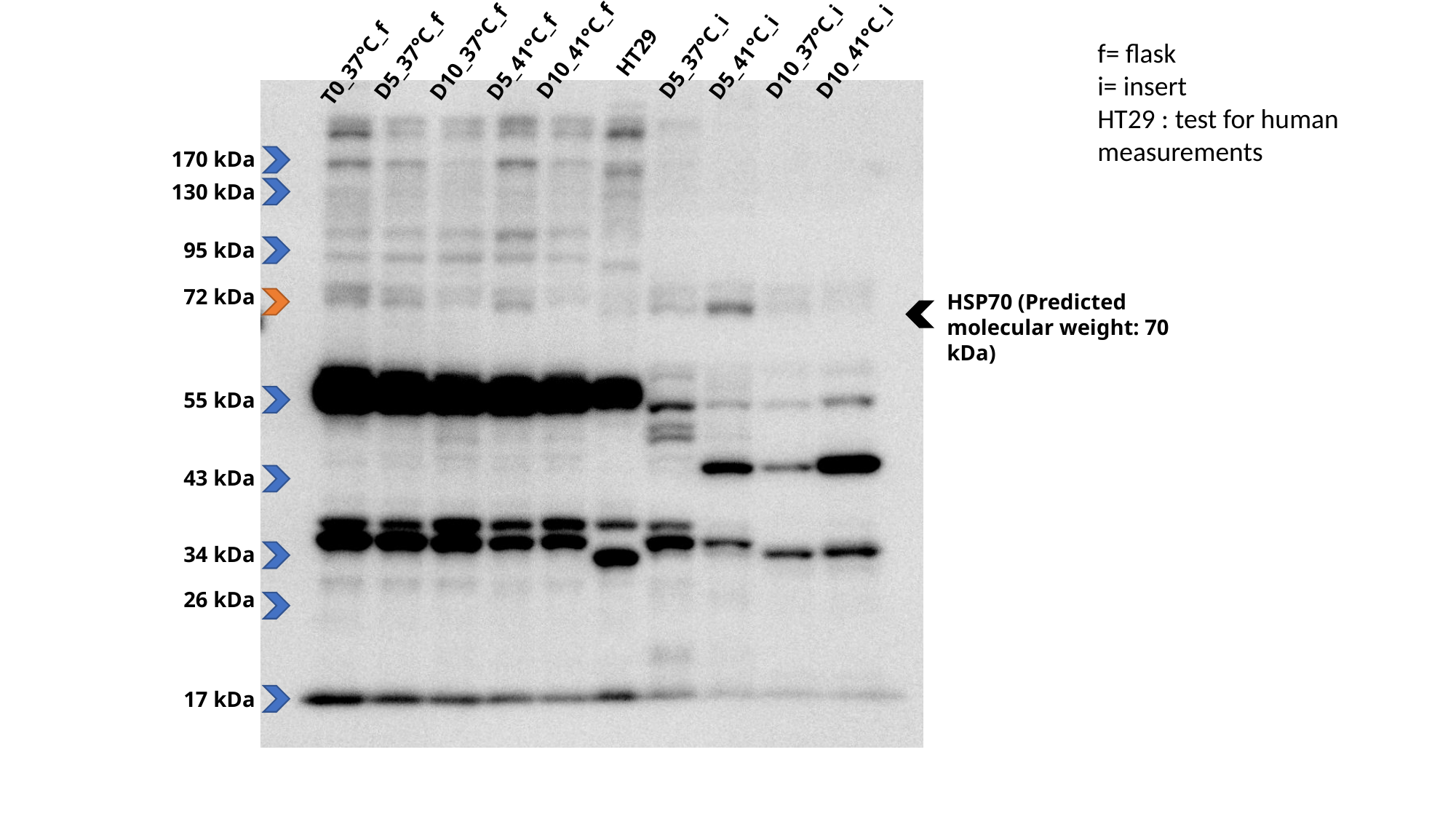

D10_41°C_f
D10_37°C_i
D10_41°C_i
D10_37°C_f
HT29
D5_37°C_f
D5_37°C_i
D5_41°C_f
D5_41°C_i
T0_37°C_f
f= flask
i= insert
HT29 : test for human
measurements
170 kDa
130 kDa
95 kDa
72 kDa
55 kDa
43 kDa
34 kDa
26 kDa
17 kDa
HSP70 (Predicted molecular weight: 70 kDa)

## Slide 6
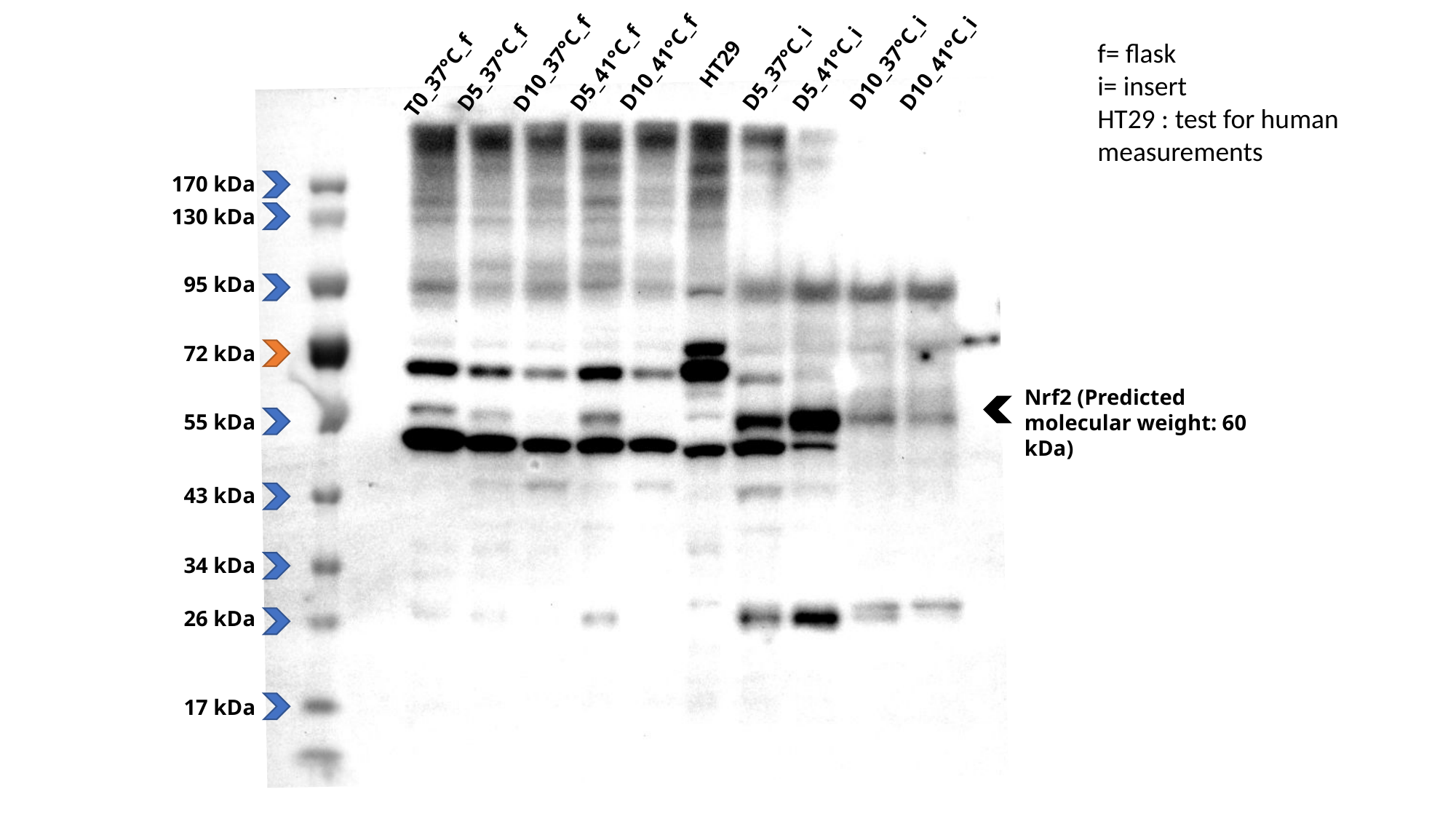

D10_41°C_f
D10_37°C_i
D10_41°C_i
D10_37°C_f
HT29
D5_37°C_f
D5_37°C_i
D5_41°C_f
D5_41°C_i
T0_37°C_f
f= flask
i= insert
HT29 : test for human
measurements
170 kDa
130 kDa
95 kDa
72 kDa
Nrf2 (Predicted molecular weight: 60 kDa)
55 kDa
43 kDa
34 kDa
26 kDa
17 kDa

## Slide 7
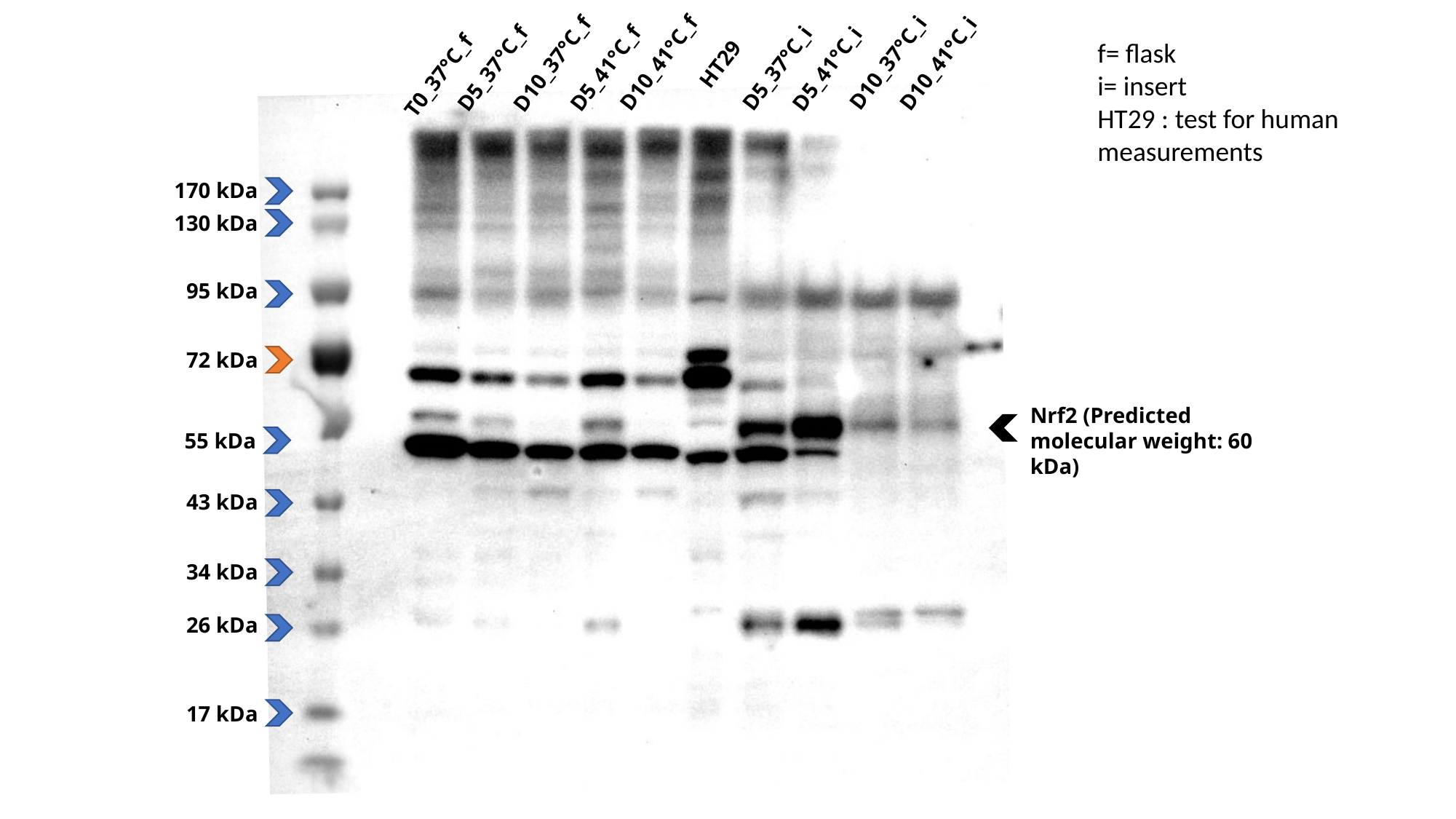

D10_41°C_f
D10_37°C_i
D10_41°C_i
D10_37°C_f
HT29
D5_37°C_f
D5_37°C_i
D5_41°C_f
D5_41°C_i
T0_37°C_f
f= flask
i= insert
HT29 : test for human
measurements
170 kDa
130 kDa
95 kDa
72 kDa
Nrf2 (Predicted molecular weight: 60 kDa)
55 kDa
43 kDa
34 kDa
26 kDa
17 kDa

## Slide 8
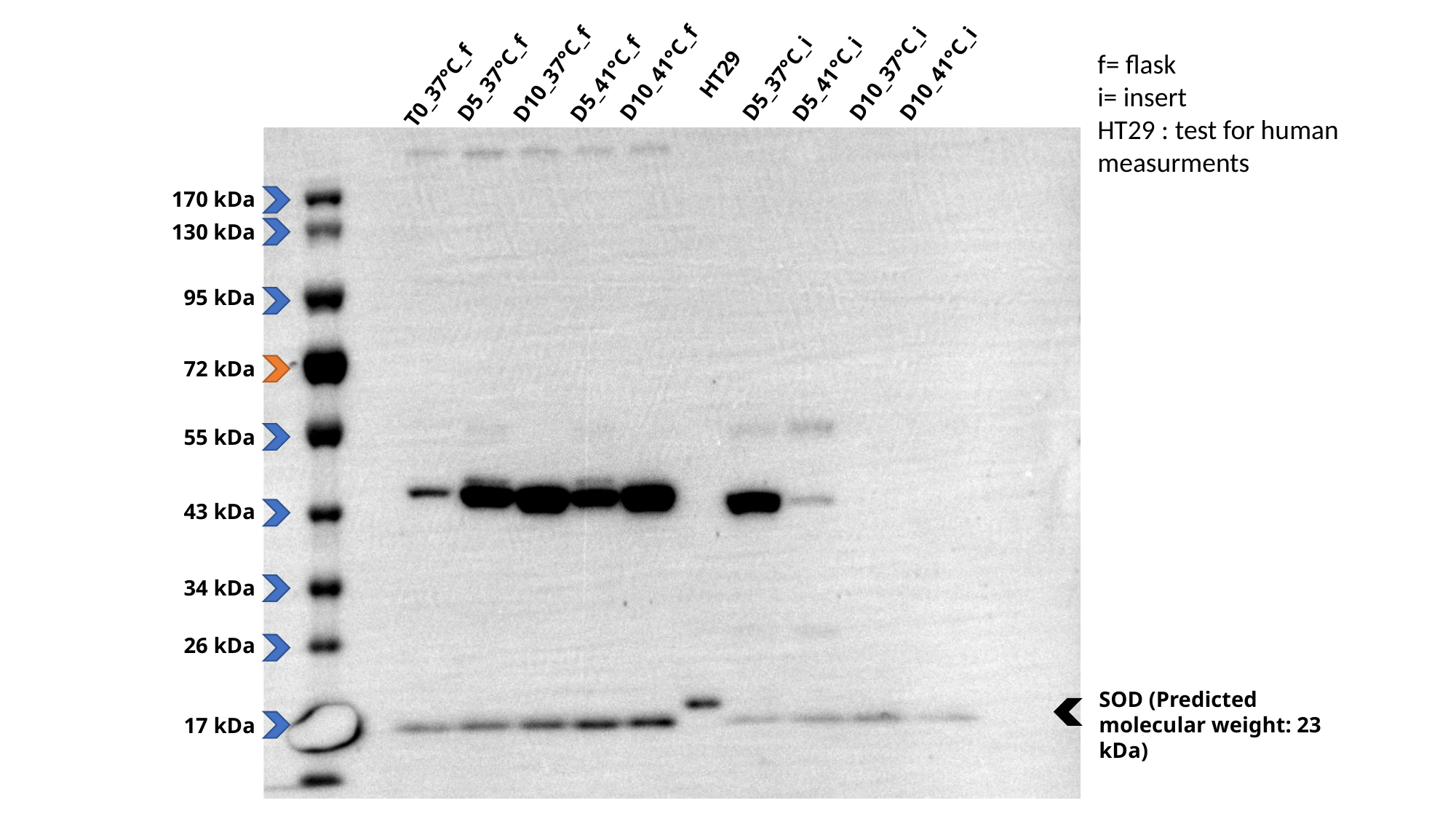

D10_41°C_f
D10_37°C_i
D10_41°C_i
D10_37°C_f
HT29
D5_37°C_f
D5_37°C_i
D5_41°C_f
D5_41°C_i
T0_37°C_f
f= flask
i= insert
HT29 : test for human
measurments
170 kDa
130 kDa
95 kDa
72 kDa
55 kDa
43 kDa
34 kDa
26 kDa
SOD (Predicted molecular weight: 23 kDa)
17 kDa
